# Non-Targeted Electronic-Nose Fingerprinting and Microscopic Imaging for Leather Classification via Transformer-Based Multimodal Fusion

**DOI:** 10.3390/molecules31213879

**Published:** 2026-10-27

**Authors:** Xiaoran Wang, Juan Chen, Yu Gu

**Affiliations:** 1 College of Information Science and Technology, Beijing University of Chemical Technology, Beijing 100029, China; 2021400222@buct.edu.cn (X.W.); jchen@mail.buct.edu.cn (J.C.); 2 School of Biomedical Engineering, Capital Medical University, Beijing 100069, China

**Keywords:** leather classification, electronic nose, non-targeted fingerprinting, microscopic imaging, multimodal data fusion

## Abstract

The rapid, non-destructive screening of leather authenticity requires analytical measurements that capture complementary volatile-response and microstructural information. This study developed RLFormer, a multimodal workflow combining cross-responsive metal-oxide-semiconductor electronic-nose (E-nose) fingerprints with microscopic surface images. Four classes—synthetic, pig, sheep, and cattle leather—were represented by 100 specimens each and measured during a standardized 10-day campaign. The relative standard deviations of the category-level daily mean E-nose responses ranged from 1.4 to 13.3% (global mean, 6.18%), and a variance-component analysis revealed a progressive between-day response attenuation common to all categories. A ResNet50 visual encoder and a two-layer long short-term memory (LSTM) encoder generated modality-specific representations that were integrated by a transformer encoder. With days 1–7 used for training and days 8–10 for testing, RLFormer achieved an accuracy of 97.67±2.16% and a Cohen’s kappa of 0.9689±0.0288 over five random seeds, compared with an 87.50±2.50% accuracy for direct ResNet50–LSTM fusion. The results demonstrate the value of combining non-targeted volatile-response patterns with microscopic texture for rapid leather classification. The workflow is intended for screening rather than compound-specific identification and requires independent validation before deployment.

## 1. Introduction

Leather is a durable, high-value material used in the fashion, automotive, furniture, and other manufacturing sectors [1,2]. Reliable distinction among animal-derived leathers and synthetic substitutes is important for quality control, market supervision, and origin verification [3]. Authentication can also support responsible sourcing and the control of illegal trade in protected species [4]. Conventional inspection relies heavily on a visual and tactile assessment by experienced personnel, but such judgments are subjective and can vary with operator experience and environmental conditions [5,6]. Objective, non-destructive analytical workflows are therefore desirable for routine screening.

Established instrumental methods provide chemically specific information. Fourier-transform infrared (FTIR) and Raman spectroscopy can characterize the molecular and structural features of leather materials [7,8,9]. These vibrational techniques are rapid and require little sample preparation, but the spectra of finished leathers can be dominated by surface coatings and dyes, and differences among animal species are subtle because the underlying collagen matrix is chemically similar. Mass-spectrometric approaches can identify species-related markers with a high specificity [3,10,11], but they usually involve destructive sampling, dedicated sample preparation or ionization sources, costly instrumentation, and specialist operation. These methods are therefore powerful reference techniques, but their requirements can limit rapid high-throughput use. An electronic nose (E-nose) offers a different analytical strategy: a cross-responsive sensor array does not identify individual volatile organic compounds (VOCs), but produces a multivariate response pattern that can be treated as a non-targeted headspace fingerprint. E-noses are inexpensive, portable, and fast, and, combined with chemometric or machine-learning methods, they have been used to discriminate material types and product origins; to monitor the state of products during ripening, storage, and spoilage; and to grade product quality and detect adulteration or counterfeiting [12,13,14,15,16]. Their main drawbacks are their limited selectivity, sensitivity to humidity and temperature, and sensor drift, which require standardized sampling, regular calibration, and appropriate pattern-recognition models [12,17]. Their suitability for leather authentication, however, has received limited systematic investigation.

Microscopic imaging provides an orthogonal source of information by describing the pore morphology, grain pattern, and surface texture. Machine-vision systems are non-contact, rapid, and relatively inexpensive and have long been used for the quality inspection of products [18]. For leather, machine-vision and deep-learning methods have been extensively investigated for defect detection and segmentation [19,20,21,22,23,24,25,26]. Digital microscopic imaging has also shown promise for leather-species classification [27,28,29,30], and convolutional neural networks have been applied to distinguish leathers produced with different vegetable tanning agents [31]. Deep-learning models learn discriminative features directly from images, but they typically require sufficient labeled data and offer limited interpretability. Moreover, an image-only analysis may be affected by illumination, finishing treatments such as embossing that imitate natural grain, within-class heterogeneity, and subtle visual similarity between classes.

Multimodal learning can integrate heterogeneous measurements that describe different aspects of the same specimen [32,33,34]. It can improve the robustness when one modality is ambiguous, at the cost of requiring paired measurements and models with more parameters. The fusion of E-nose and computer-vision data has been reported for food quality assessments, for example, in tea-quality identification and meat-freshness evaluations [35,36,37]. For leather analyses, the combination of non-targeted headspace fingerprints and the microscopic texture is particularly attractive because the two modalities are analytically complementary: the E-nose records dynamic, cross-responsive volatile patterns, whereas microscopy records the surface microstructure. To the best of our knowledge, however, this combination has not been systematically investigated for leather classification, and the analytical value of interaction-based fusion under a held-out-day evaluation remains unclear.

Accordingly, this study treated the E-nose signals as class-dependent multichannel fingerprints and tested the hypothesis that these fingerprints and microscopic images provide complementary information, allowing interaction-based fusion to outperform either modality alone and simple feature concatenation. The study objectives were to (i) establish a standardized 10-day paired acquisition workflow; (ii) characterize the category-level between-day dispersion and its variance components, sensor-wise discrimination, and inter-sensor correlation; (iii) develop RLFormer, which combines modality-specific encoders with transformer-based cross-modal interaction; and (iv) evaluate the models on specimens acquired during held-out later days. Compound-specific assignments were not made from the cross-responsive sensor data.

## 2. Results and Discussion

### 2.1. Dynamic E-Nose Fingerprint Characteristics

**Figure 1:**
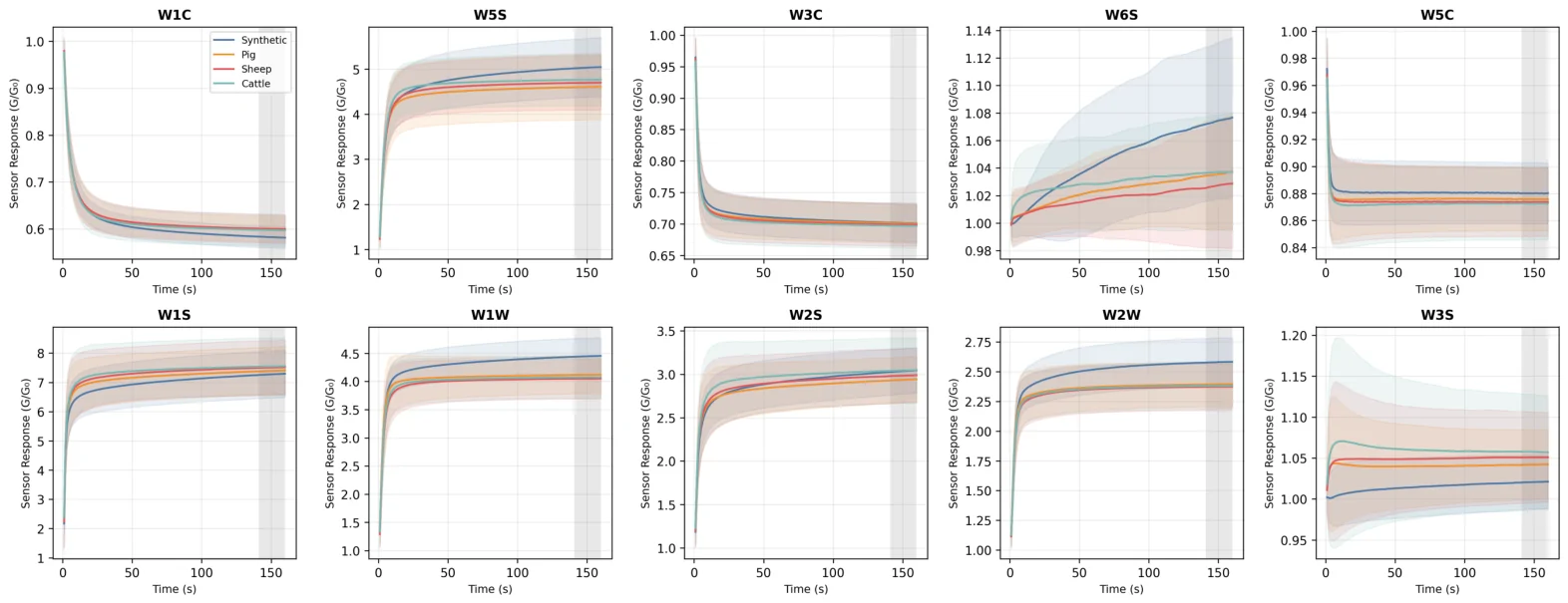
Dynamic response curves ($G/G_{0}$) of the 10 PEN3 sensors for the four leather categories. Shaded regions indicate the standard deviation, and the gray band denotes the fixed window (141–160 s) used for response summaries.

The four leather categories produced distinct mean temporal response profiles across the 10 MOS channels (Figure 1). Several channels showed class-dependent divergence during the transient stage that remained visible in the 141–160 s fixed window. W1W, W2W, and W6S displayed comparatively pronounced separation, whereas other channels showed greater overlap. These observations indicate that the array recorded class-dependent multivariate headspace patterns suitable for a subsequent pattern-recognition analysis. They do not, by themselves, establish the identity or concentration of individual volatile compounds.

The responses within the fixed window were quasi-stationary for most channels, but did not represent a true steady state for all of them. For eight sensors (W1C, W5S, W3C, W5C, W1S, W1W, W2S, and W2W), the mean absolute relative change between 141 and 160 s was only 0.14–0.52% across the 400 specimens, corresponding to 0.4–1.5% of the total response excursion over the measurement. For seven of these sensors, the median rate of change in the final window decreased to 9–18% of that in the 21–40 s window; W5C changed little throughout the measurement. In contrast, W6S and W3S, whose responses deviated from the baseline by only about 4% ($G/G_{0}\approx 1.04$), had not reached a plateau; for W6S, the median rate of change in the final window remained 77% of that in the early window. The 141–160 s interval is therefore described as a fixed-time window rather than as a steady state. Because all specimens were measured under identical flow and timing conditions, responses in this window nevertheless provide a standardized basis for comparison among specimens.

### 2.2. Between-Day Dispersion and Sensor-Level Information

**Figure 2:**
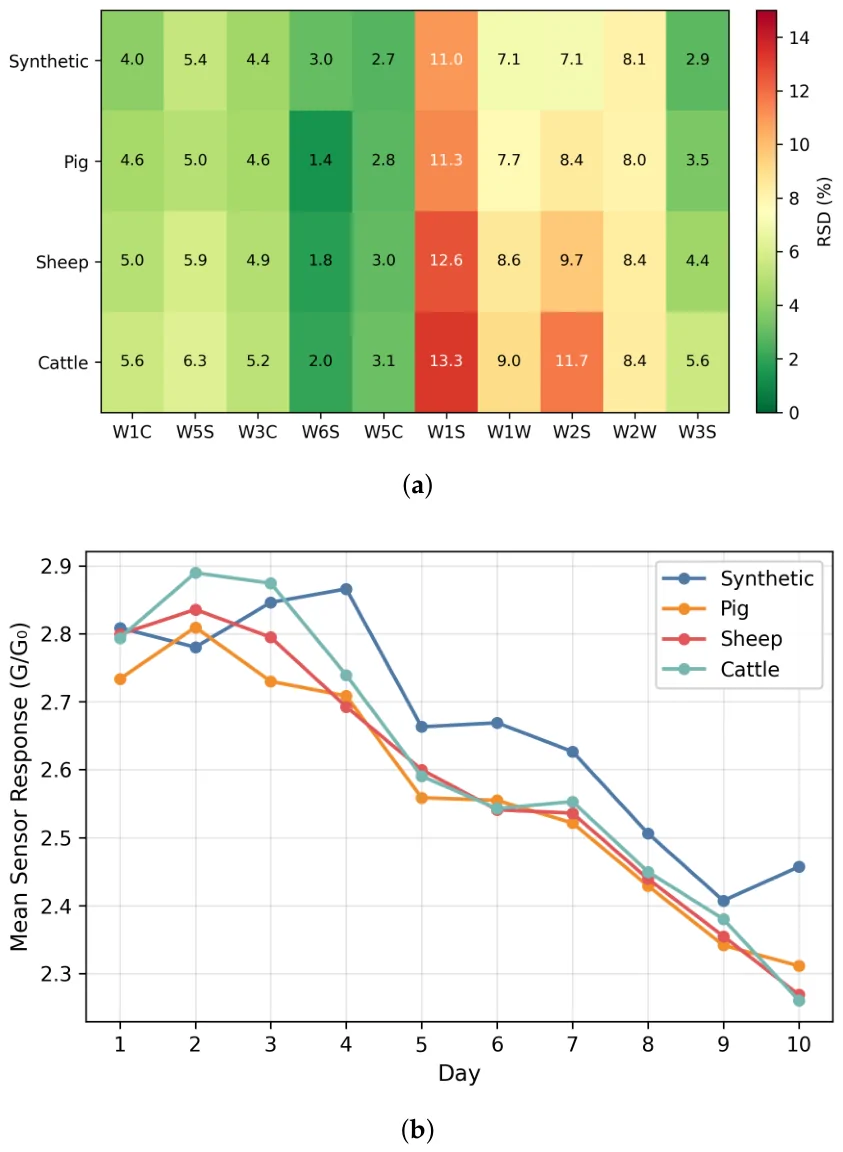
Between-day dispersion of E-nose fixed-window responses during the 10-day acquisition period: (**a**) RSD heatmap of the category-level daily mean responses; (**b**) daily mean response of each leather category averaged across all 10 sensors.

The RSD values of the category-level daily mean fixed-window responses ranged from 1.4% to 13.3%, with a global mean of 6.18% (Figure 2a). W6S and W5C showed the lowest dispersion (≤3.1%), whereas W1S showed the largest, reaching 13.3% for cattle leather. The daily means, however, did not fluctuate randomly around a constant level. Averaged across the 10 sensors, the responses of all four categories decreased in parallel over the campaign, by 12.5–19.1% from day 1 to day 10 (Figure 2b).

The variance-component analysis clarified the origin of this dispersion (Table 1). For most channels, the between-day variation exceeded the within-day variation: averaged over the categories, ${\mathrm{RSD}}_{d}$ ranged from 1.5% (W6S) to 12.0% (W1S), and acquisition day accounted for 65–95% of the total variance (ICC = 0.65–0.95) for eight of the ten sensors. The within-day RSD values, which include the specimen heterogeneity and therefore bound the measurement repeatability from above, were below 3.5% for six sensors (W1C, W3C, W5C, W1S, W2W, and W3S) and averaged 3.81% across all sensor–category combinations. W5S and W6S behaved differently: their variability was dominated by within-day differences (ICC = 0.10 and 0.12, respectively), and W5S showed the largest within-day RSD (12.9%).

Significant linear trends with acquisition day were detected for 31 of the 40 sensor–category combinations (p<0.05). The direction of these trends corresponded to a progressive attenuation of the response magnitude: channels with responses above unity (W5S, W1S, W1W, W2S, and W2W) decreased by 1.4–3.9% per day, whereas channels with responses below unity (W1C, W3C, and W5C) increased toward unity by 0.9–1.5% per day. The trends were of similar magnitude in all four categories, including the synthetic material, which differs chemically from the three animal-derived leathers. Because specimen selection was not associated with acquisition day, this common, monotonic attenuation is most consistent with time-dependent sensor drift and/or slow changes in the measurement environment, although gradual changes in the stored leather stock cannot be entirely excluded. As a consequence, the test specimens acquired on days 8–10 produced mean responses 10.7–12.8% lower than those of the training period, so the chronological partition evaluates classification under realistic short-term drift rather than under stationary conditions.

**Figure 3:**
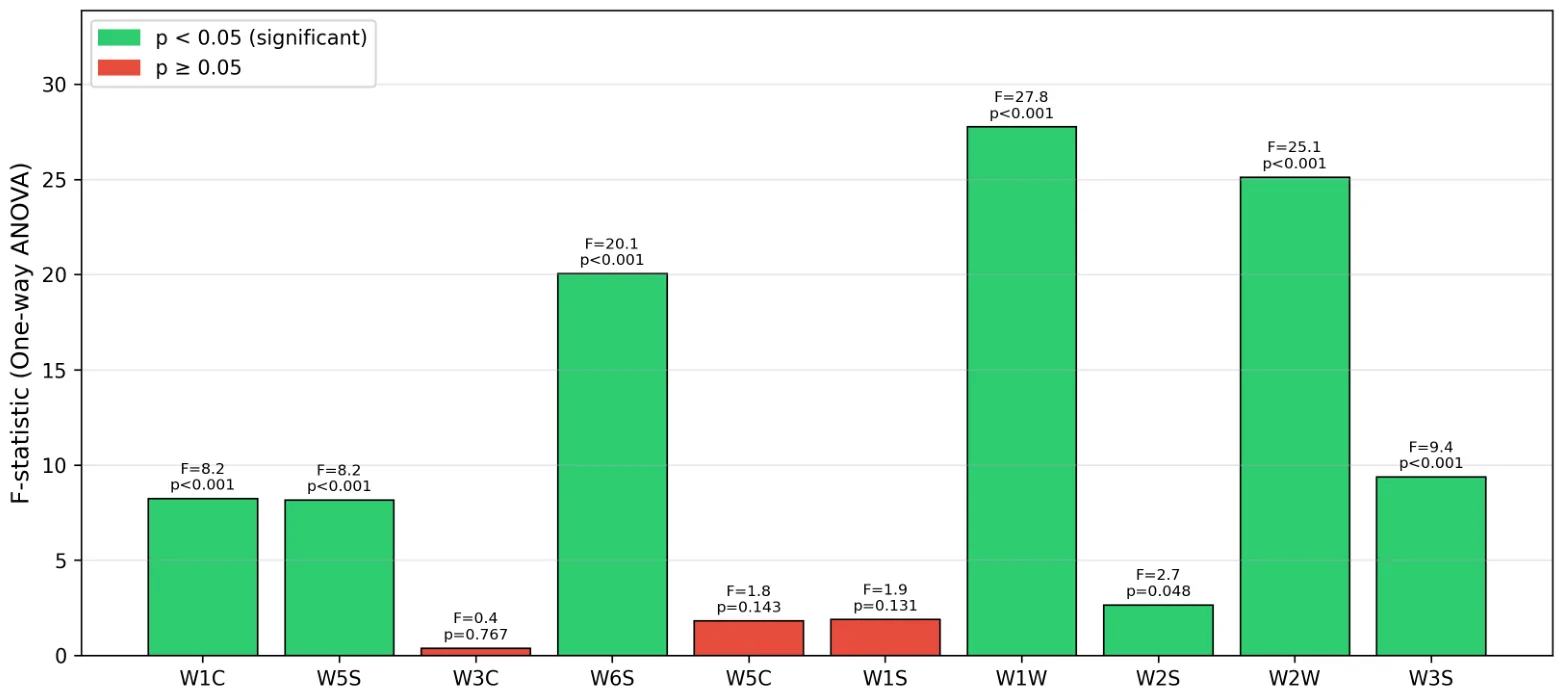
Exploratory one-way ANOVA F-statistics of the 10 PEN3 sensors for distinguishing the four leather categories using fixed-window responses. Green bars indicate p<0.05, and red bars indicate p≥0.05.

The exploratory one-way ANOVA showed substantial differences in sensor-wise class separation (Figure 3). W1W, W2W, and W6S had the largest F-statistics (F=27.77, 25.12, and 20.06, respectively; p<0.001). Seven of the ten sensors differed significantly among the four categories (p<0.05), although W2S did so only marginally (p=0.048), whereas W3C, W5C, and W1S did not show statistically detectable differences (p>0.05). The result indicates that a subset of channels contained strong class-related information, while the remaining channels could still contribute complementary temporal or interaction patterns in multivariate models.

**Figure 4:**
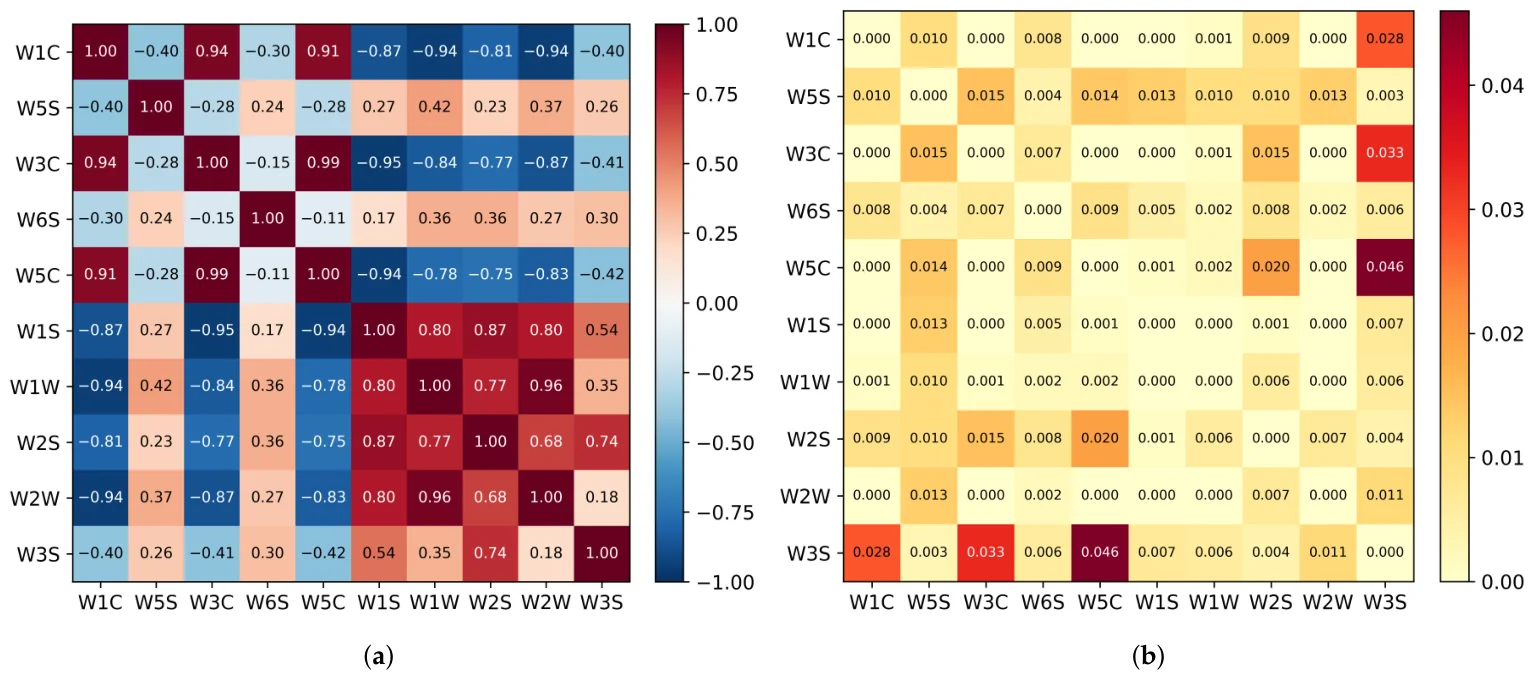
Inter-sensor correlation analysis: (**a**) Pearson correlation matrix of the 10 sensors across all 400 specimens; (**b**) variance of inter-sensor correlation across leather categories.

The correlation matrix revealed partial redundancy within the array (Figure 4a). W1W and W2W were strongly positively correlated (r>0.95), indicating closely covarying response patterns. W6S and W3C showed weaker correlations with several other channels and therefore contributed more independent response dimensions. The category-wise variance of the correlation coefficients was generally low, although pairs involving W3S and W5C varied more across categories (Figure 4b). Thus, the array combined correlated and complementary channels, providing a multidimensional fingerprint rather than 10 fully independent measurements.

The nominal sensitivity labels of the PEN3 sensors (Section 3.2) can motivate hypotheses about broad chemical families, but the present data do not permit compound assignment. Cross-responsive MOS signals can be affected by co-occurring compounds, concentration, humidity, and matrix effects. Here, matrix effects refer to the influence of the leather matrix itself—collagen fibres, tanning agents, fatliquors, dyes, finishing coatings, polyurethane layers in the synthetic material, and moisture—on the release of volatiles into the headspace and on the sensor response, for example, through background emissions, water vapour, and competitive adsorption on the sensing surface, which can modify the signal of a given compound non-specifically. Accordingly, the observed W1W, W2W, and W6S differences are interpreted as discriminative response patterns rather than direct evidence for specific sulfur compounds, aromatics, or hydrogen. Future coupling with a compound-resolving reference technique would be required to identify the volatile markers underlying the classification, as demonstrated in analytical workflows that combine E-nose screening with GC–MS [16].

### 2.3. Exploratory PCA and Conventional E-Nose Classification

**Figure 5:**
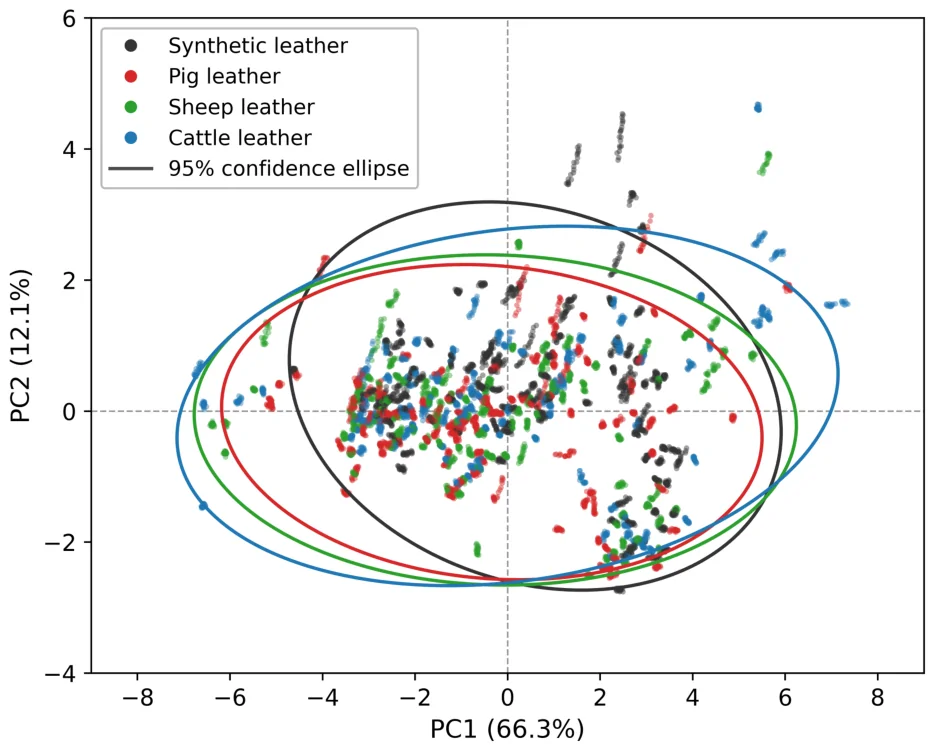
PCA score plot for the four leather categories based on the E-nose fixed-window responses. Each point represents one 1 s observation within the 141–160 s window. Ellipses indicate the 95% confidence regions of each category.

The first two principal components explained 66.3% and 12.1% of the total variance, respectively, for a cumulative 78.4% (Figure 5). The score plot showed partial separation between synthetic and natural leather along PC1, but the four classes were not fully resolved in two dimensions. The PCA therefore confirmed that the E-nose feature space contained class-related structure while also showing that linear projection alone was insufficient for complete discrimination.

The conventional E-nose classifiers produced a moderate and model-dependent performance (Table 2). SVM achieved the best results, with a 70.92% accuracy, a weighted F1-score of 70.17%, and a kappa of 0.6122. RF and KNN achieved accuracies of 50.46±3.01% and 34.75%, respectively. These results show that the E-nose fingerprint alone contained useful class information, but that the selected conventional models did not fully capture the structure of the sensor responses under the held-out-day evaluation.

### 2.4. Microscopic-Image Classification

The image-only models produced a moderate and seed-dependent performance (Table 3). The basic CNN achieved the highest mean accuracy (82.83±8.69%) and weighted F1-score (81.99±9.98%), followed by ResNet50 (77.33±6.86%) and VGG16 (66.67±8.29%). All three networks showed noticeable variation across seeds (standard deviations of 6.9–8.7 percentage points). The simpler CNN may have been better matched to the limited training-set size and texture-focused task, whereas the higher-capacity networks offered no improvement in the mean accuracy when trained on 280 images. As illustrated by the representative images in Section 3.1, specimens within the same category also differed in colour and local texture; because the images were converted to grayscale, colour differences did not contribute directly to classification. The results confirm that microscopic texture is informative, but does not fully resolve the four classes by itself.

### 2.5. Multimodal Fusion and Ablation Analysis

The ablation study demonstrated a clear benefit from combining the two measurement modalities (Table 4). The ResNet50 and LSTM unimodal baselines achieved mean accuracies of 77.33±6.86% and 62.50±2.50%, respectively. Adding a transformer after a single-modality encoder increased the mean accuracy to 79.67±3.26% for ResNet50 + transformer and 77.67±2.24% for LSTM + transformer. The direct fusion of the ResNet50 and LSTM embeddings achieved 87.50±2.50%, confirming that microscopic and E-nose information were complementary.

The standalone transformer applied to fused features without dedicated modality-specific encoders achieved 82.00±6.89%, lower than direct ResNet50 + LSTM fusion, indicating that attention alone did not replace appropriate visual and temporal feature extraction. RLFormer, which combined both modality-specific encoders and transformer-based interaction, achieved the highest mean performance: a 97.67±2.16% accuracy, a 97.83±1.89% weighted precision, a 97.67±2.16% weighted recall, a 97.65±2.17% weighted F1-score, and a kappa of 0.9689±0.0288. Relative to direct ResNet50 + LSTM fusion, the mean accuracy increased by 10.17 percentage points, and RLFormer also showed the smallest standard deviation among the multimodal configurations.

**Figure 6:**
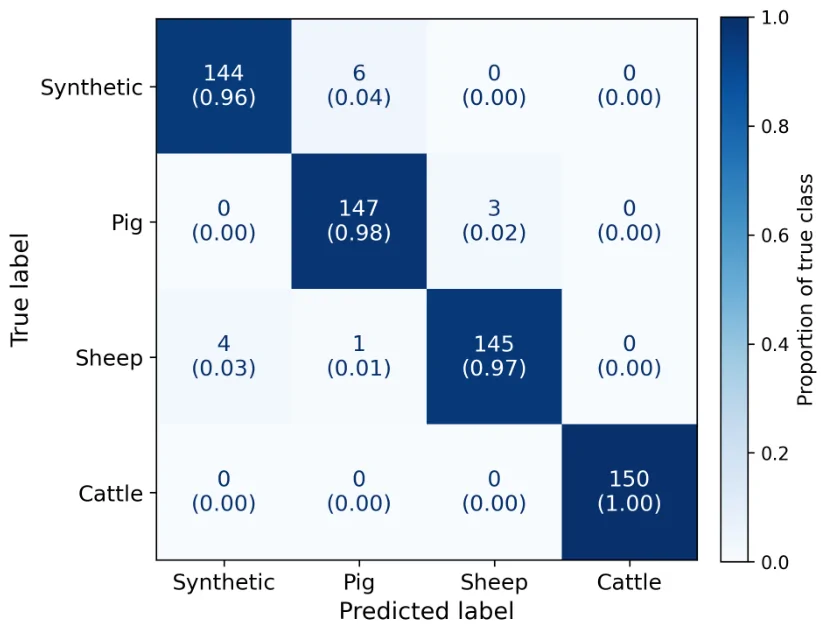
Confusion matrix of RLFormer on the chronological test set, aggregated over five random seeds (600 predictions; 150 per category).

Figure 6 summarizes the RLFormer predictions aggregated over the five seeds (600 test predictions). Cattle leather was classified correctly in all 150 cases. The 14 misclassifications were distributed among the other three categories: six synthetic-leather predictions as pig leather, three pig-leather predictions as sheep leather, four sheep-leather predictions as synthetic leather, and one sheep-leather prediction as pig leather. These results indicate a strong performance on the held-out acquisition days of the present campaign, even though the E-nose response levels on these days were systematically lower than those of the training period (Section 2.2).

The reported values were obtained with five random seeds on one fixed chronological split. The standard deviations therefore reflect variability due to model initialization, but not variability due to data partitioning. The study did not include nested model selection or an external test set. The results therefore support the proposed fusion strategy within the present acquisition campaign, but do not establish statistical superiority across laboratories, instruments, leather lots, or operating environments.

### 2.6. Analytical Implications, Limitations, and Future Validation

From an analytical perspective, the principal finding is that a non-targeted, cross-responsive headspace fingerprint and a microscopic structural measurement provide complementary information. The E-nose subsystem alone and the image subsystem alone achieved moderate accuracies, whereas their integration substantially improved the classification performance. The interaction-based fusion further improved on direct concatenation, suggesting that the relationship between the modalities was informative rather than merely additive. In this type of array-based analysis, selectivity is achieved at the level of the multichannel response pattern and its data-analysis model rather than by a compound-specific sensor. The proposed workflow is therefore best positioned as a rapid screening approach; samples with regulatory, forensic, or high-value consequences would require confirmation by a chemically specific reference method. In practical terms, once the model has been trained, classification proceeds by end-to-end inference: an operator acquires an E-nose record and a microscopic image according to the documented protocol, and the category is predicted without manual feature engineering or data interpretation. The instrumentation required, a portable E-nose and a handheld digital microscope, is also considerably less demanding than chromatographic or mass-spectrometric equipment.

The between-day attenuation observed in this study also has practical implications for routine operation. Over the 10-day campaign, the mean response decreased by 12.5–19.1%. Although RLFormer remained accurate on the later acquisition days, longer deployment would require explicit drift management. Practical options include (i) measuring a reference material or calibration gas at the start of each session to monitor sensor sensitivity and rescale the responses, as recommended by the instrument manufacturer for long measurement series [38]; (ii) normalizing the response patterns relative to such daily references, so that classification relies on relative rather than absolute response magnitudes; (iii) applying established multivariate drift-correction methods, such as component correction [17]; and (iv) periodically updating the model with a small number of newly acquired labeled specimens through domain adaptation, fine-tuning, or transfer learning [39]. Implementing and validating these procedures over longer periods will be part of future work.

Several limitations define the interpretation of these results. First, the PEN3 array does not identify individual VOCs; GC–MS, GC–IMS, or another compound-resolving method is needed to associate discriminative sensor patterns with chemical markers. Second, the variance-component analysis separated within-day and between-day contributions, but it was based on different specimens rather than replicate measurements of the same specimen; ${\mathrm{RSD}}_{w}$ is therefore an upper bound on repeatability, and the origin of the between-day attenuation (sensor drift versus gradual changes in the stored leather stock) could not be resolved without reference measurements. Third, the dataset comprised 400 specimens from four categories obtained from a single commercial supplier and collected during one controlled 10-day campaign, so broader generalization remains to be established across independent hides or production lots, finishing and tanning conditions, instruments, sites, and external test data. Fourth, the five-seed evaluation quantified the variability due to model initialization, but not the variability due to data partitioning, because only one chronological split was used. Fifth, W6S and W3S had not reached a plateau within the fixed window, so the features derived from these channels depend more strongly on the exact sampling time. Finally, the external reproducibility depends on access to the original data and code together with complete software, preprocessing, and model-configuration details.

Future studies should combine repeated quality-control measurements of reference materials with multi-lot sampling, use compound-resolving analyses to characterize the volatile contributors, evaluate the robustness to humidity and environmental variation, and perform external validation on independently sourced specimens. These steps will determine whether the present proof of concept can be translated into a reliable screening method for routine leather authentication.

## 3. Materials and Methods

### 3.1. Study Design and Leather Specimens

**Figure 7:**
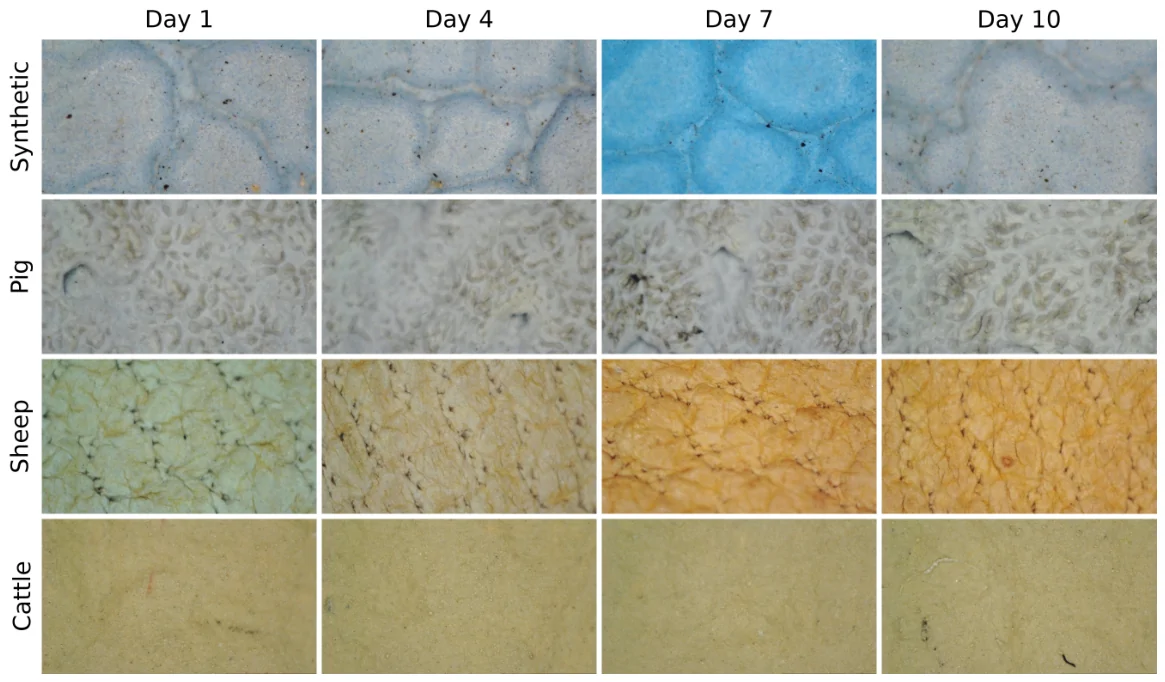
Representative microscopic images (50×) of the four leather categories. Each row corresponds to one category (synthetic, pig, sheep, and cattle leather), and each column corresponds to one acquisition day (days 1, 4, 7, and 10). Each panel shows a different specimen; specimens were selected by stratified random sampling within each category–day stratum. Images are shown as acquired, without cropping or colour adjustment. Differences in colour among panels of the same category reflect natural specimen-to-specimen variation; images were converted to grayscale before model input.

A total of 400 leather specimens were included, with 100 specimens in each of four categories: synthetic leather, pig leather, sheep leather, and cattle leather. All four leather materials were commercially purchased, processed leather materials obtained from Yantai Tannery Co., Ltd. (Yantai, Shandong, China). No live animals were involved in this study. The specimens were prepared and selected under the guidance of experienced leather experts to maintain consistency in the material category and surface integrity. On each measurement day, specimens (approximately 20×10 mm) were freshly cut from the stock material of each category, selected without a predefined order, and numbered according to the measurement sequence. Apart from cutting to size, no chemical or destructive pretreatments were applied. A paired E-nose record and microscopic image were obtained from each specimen. For the headspace measurement, each specimen could be accommodated in a 50 mL sealed vial without altering the original sample surface. Representative microscopic images of specimens from each category and from different acquisition days are shown in Figure 7.

The measurements were conducted over 10 consecutive days. On each day, 40 specimens were analyzed, comprising 10 specimens from each category. Because the specimens were freshly prepared and selected without a predefined order on each day, specimen heterogeneity was not systematically associated with the acquisition day. E-nose measurements were performed in the morning, followed by microscopic imaging in the afternoon. The acquisition day was retained as the grouping variable for the chronological train–test partition described in Section 3.6.

### 3.2. Electronic-Nose Headspace Acquisition

**Figure 8:**
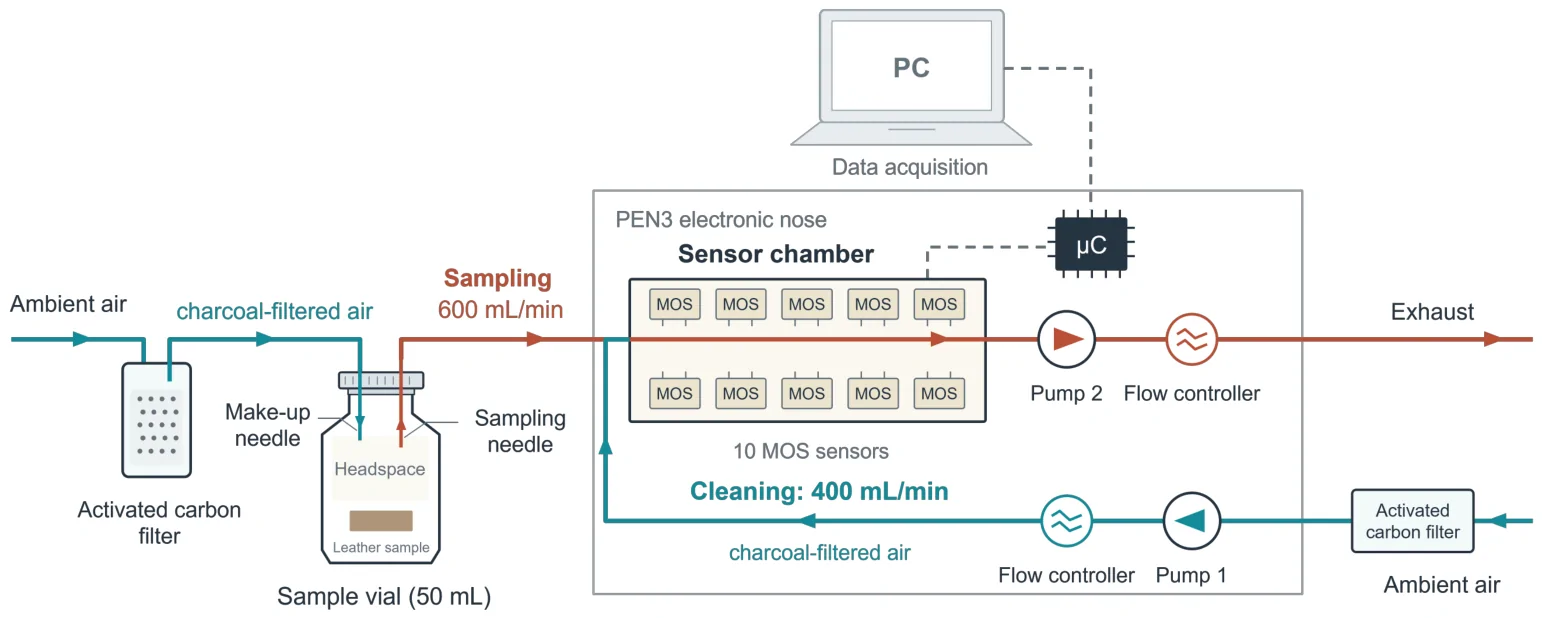
Schematic of the headspace sampling and gas-flow configuration of the PEN3 electronic nose. The leather specimen (approximately 20×10 mm) was placed in a 50 mL sealed vial fitted with a sampling needle and a make-up needle. During the sampling stage (160 s), the headspace was drawn through the sensor chamber at 600 mL/min while charcoal-filtered air entered the vial through the make-up needle to replace the withdrawn volume; the vial therefore operated as a dynamic headspace rather than as a static equilibrium system. Charcoal-filtered ambient air was used for the 5 s zero-point adjustment and for flushing the sensor chamber at 400 mL/min for 30 s between measurements.

E-nose data were acquired using a PEN3 system (Airsense Analytics GmbH, Schwerin, Germany) equipped with 10 metal-oxide-semiconductor (MOS) sensors. Headspace sampling followed the manual dynamic headspace configuration described by the manufacturer [38], as illustrated in Figure 8. Each specimen was placed in a 50 mL sealed vial and equilibrated for 3 min at room temperature without heating to promote the accumulation of volatile constituents in the headspace. Two needles were then inserted through the seal of the vial: a sampling needle connected to the inlet of the instrument and a make-up needle connected to an activated charcoal filter, through which filtered air entered the vial to replace the withdrawn headspace. The measurements were performed in a clean, well-ventilated laboratory at 24±2 °C and 50±2% relative humidity. Laboratory ambient air passed through activated charcoal was used as zero gas, and the automatic dilution (autoranging) function of the instrument was deactivated, so that no zero gas was added to the sampled headspace (dilution factor = 1).

The measurement cycle comprised three stages. First, a 5 s zero-point adjustment was performed with zero gas to establish the baseline conductance $G_{0}$ of each sensor. Second, vial headspace was drawn through the sensor chamber at 600 mL/min for 160 s, and the 10 sensor responses were recorded at 1 Hz and expressed as the relative conductance $G/G_{0}$. Third, the sensor chamber was flushed with charcoal-filtered air at 400 mL/min for 30 s before the next measurement. Because the volume of gas drawn during the 160 s sampling stage (approximately 1600 mL) greatly exceeded the vial volume, the initial headspace was progressively replaced by filtered make-up air, and the vial operated as a dynamic headspace system rather than as a static equilibrium system. The recorded signal therefore reflected the combined contribution of the initially accumulated headspace and the continued release of volatiles from the specimen under constant flow. Because all specimens were measured with the same specimen size, equilibration time, flow rates, and timing, the resulting response curves were treated as standardized dynamic fingerprints. The same protocol was used throughout the 10-day campaign.

The nominal sensitivity classes of the 10 MOS sensors are summarized in Table 5. These broad manufacturer-defined response tendencies are useful for describing the array, but they do not imply compound-specific selectivity. Consequently, responses from an individual channel were not interpreted as proof of the presence or concentration of a particular VOC.

### 3.3. Microscopic Image Acquisition

Images were acquired using a Celestron Handheld Digital Microscope Pro (Celestron, Torrance, CA, USA) equipped with a 5 MP CMOS sensor and an integrated upper-ring illuminator comprising eight adjustable white-light-emitting diodes. Imaging was performed in a conventional reflected-light configuration under visible white-light illumination; ultraviolet and hyperspectral imaging were not used. The acquired RGB images therefore provided spatial information on the visible surface appearance and texture of the specimens rather than wavelength-resolved spectral information. Each specimen was imaged at 50× magnification in the central surface region at a resolution of 1280×720 pixels. Magnification, illumination, and the imaging procedure were held constant throughout the campaign. Representative images are shown in Figure 7.

### 3.4. E-Nose Response Summaries and Exploratory Statistical Analysis

The full 160 s response sequence of each specimen was used as input to the LSTM-based models (Section 3.7). For the statistical analyses, PCA, and conventional classifiers, the responses were summarized within a fixed time window. For each specimen and sensor channel, the fixed-window response was defined as the mean $G/G_{0}$ over 141–160 s. To verify that this window represented a quasi-stationary portion of the response, the relative change in each sensor response between 141 and 160 s was calculated for every specimen, together with the ratio between the rate of change in this window and that in the 21–40 s window, where the rate of change was estimated as the least-squares slope normalized by the window mean.

For each category and acquisition day, the daily mean was calculated from the 10 specimens measured for that category on that day. The between-day dispersion of these category-level daily means was summarized using the relative standard deviation (RSD):

(1) $$\mathrm{RSD}\;\left(\%\right)=\frac{\sigma }{\mu }\times 100,$$

where μ and σ denote the mean and the sample standard deviation (n−1 denominator), respectively, of the 10 daily category means. Because different specimens were measured on different days, this quantity combines the between-specimen heterogeneity and the between-day variation. To separate these contributions, a one-way random-effects ANOVA with acquisition day as the random factor (10 days × 10 specimens) was applied to the fixed-window response of each sensor within each category. The within-day variance and the between-day variance component were estimated as

(2) $$s_{w}^{2}={\mathrm{MS}}_{\mathrm{within}},\;s_{d}^{2}=\max\;\left(0,\;\frac{{\mathrm{MS}}_{\mathrm{between}}-{\mathrm{MS}}_{\mathrm{within}}}{n}\right),$$

where ${\mathrm{MS}}_{\mathrm{within}}$ and ${\mathrm{MS}}_{\mathrm{between}}$ are the within-day and between-day mean squares, and n=10 is the number of specimens per day. The corresponding relative standard deviations, ${\mathrm{RSD}}_{w}=s_{w}/\bar{x}\times 100$ and ${\mathrm{RSD}}_{d}=s_{d}/\bar{x}\times 100$, were expressed relative to the category mean $\bar{x}$, and the combined variability was expressed as ${\mathrm{RSD}}_{I}={\left(s_{w}^{2}+s_{d}^{2}\right)}^{1/2}/\bar{x}\times 100$. The proportion of variance attributable to acquisition day was summarized by the intraclass correlation coefficient:

(3) $$\mathrm{ICC}=\frac{s_{d}^{2}}{s_{w}^{2}+s_{d}^{2}}.$$

Because ${\mathrm{RSD}}_{w}$ was estimated from different specimens measured on the same day, it includes natural specimen heterogeneity and therefore represents an upper bound on the measurement repeatability rather than a repeatability estimate based on replicate measurements of the same specimen. Linear trends in the daily means were tested by ordinary least-squares regression on acquisition day and expressed as a percentage of the category mean per day.

An exploratory one-way analysis of variance (ANOVA) was applied separately to the fixed-window responses of each sensor (100 specimens per category) to assess differences among the four leather categories, with p<0.05 considered statistically significant. Pearson correlation coefficients were calculated between sensor channels across the 400 specimens to examine redundancy and complementarity within the array. A principal component analysis (PCA) was used as an unsupervised visualization of the E-nose feature space [40]; each 1 s observation within the 141–160 s window was treated as one 10-dimensional data point.

### 3.5. Performance Metrics

The classification performance was evaluated using the accuracy, the weighted precision, the weighted recall, the weighted F1-score, and Cohen’s kappa coefficient. Because the task comprised four equally represented classes, the weighted metrics summarize the class-specific performance while retaining the contribution of each class.

The accuracy was defined as

(4) $$\mathrm{Accuracy}=\frac{1}{N}\sum_{i=1}^{N}1\left({\hat{y}}_{i}=y_{i}\right),$$

where *N* is the number of test samples (specimens for the deep-learning models and 1 s observations for the conventional classifiers), $y_{i}$ is the true label, ${\hat{y}}_{i}$ is the predicted label, and 1(·) is the indicator function. For each class *c*, the precision, recall, and F1-score were defined as

(5) $${\mathrm{Precision}}_{c}=\frac{{\mathrm{TP}}_{c}}{{\mathrm{TP}}_{c}+{\mathrm{FP}}_{c}},$$

(6) $${\mathrm{Recall}}_{c}=\frac{{\mathrm{TP}}_{c}}{{\mathrm{TP}}_{c}+{\mathrm{FN}}_{c}},$$

(7) $$F1_{c}=\frac{2\times {\mathrm{Precision}}_{c}\times {\mathrm{Recall}}_{c}}{{\mathrm{Precision}}_{c}+{\mathrm{Recall}}_{c}},$$

where ${\mathrm{TP}}_{c}$, ${\mathrm{FP}}_{c}$, and ${\mathrm{FN}}_{c}$ denote the numbers of true positives, false positives, and false negatives for class *c*, respectively. The weighted averages were calculated as

(8) $${\mathrm{Precision}}_{w}=\sum_{c=1}^{C}\frac{n_{c}}{N}{\mathrm{Precision}}_{c},$$

(9) $${\mathrm{Recall}}_{w}=\sum_{c=1}^{C}\frac{n_{c}}{N}{\mathrm{Recall}}_{c},$$

(10) $$F1_{w}=\sum_{c=1}^{C}\frac{n_{c}}{N}F1_{c},$$

where *C* is the number of classes and $n_{c}$ is the number of test samples in class *c*. Cohen’s kappa coefficient was calculated as

(11) $$\kappa =\frac{p_{o}-p_{e}}{1-p_{e}},$$

where $p_{o}$ is the observed agreement and $p_{e}$ is the agreement expected by chance.

### 3.6. Dataset Partition, Baseline Models, and Training Procedure

The 400 paired observations were partitioned chronologically by acquisition day. The 280 specimens measured during days 1–7 formed the training set, and the 120 specimens measured during days 8–10 formed the test set. Each partition therefore contained an equal representation of the four categories. No specimen appeared in both partitions, and the image and E-nose measurement from each specimen remained paired. This fixed split was designed to test the transfer to later acquisition days within the same 10-day campaign rather than the performance under a random within-day split.

Three conventional classifiers—support vector machine (SVM), random forest (RF), and *k*-nearest neighbors (KNN)—were evaluated using the E-nose responses within the 141–160 s window. For these classifiers, each 1 s observation within the window was treated as one 10-dimensional input vector, giving 20 observations per specimen (5600 training and 2400 test observations). All observations from a given specimen were assigned to the same partition, so that no specimen contributed to both training and testing. Three image-only convolutional models—a basic CNN, VGG16, and ResNet50—were evaluated on the microscopic images. The multimodal ablation study compared ResNet50, LSTM, ResNet50 + transformer, LSTM + transformer, direct ResNet50 + LSTM fusion, a standalone transformer applied to fused features, and the complete RLFormer model. All reported configurations were evaluated using the same chronological partition and the metrics defined in Section 3.5.

The basic CNN used for the image-only comparison comprised three 3×3 convolutional layers with 32, 64, and 128 filters, respectively. Each convolution used unit padding and a rectified linear unit activation and was followed by 2×2 max pooling. The flattened feature map was passed through a 256-unit fully connected layer, a dropout layer with a rate of 0.5, and a four-class output layer. The basic CNN and RLFormer were trained for 100 epochs using the Adam optimizer, an initial learning rate of $1\times 10^{-4}$, a batch size of 32, and categorical cross-entropy loss.

For all models involving random initialization or random sampling (RF and the neural networks), training and evaluation were repeated with five random seeds (42–46) on the same chronological partition, and the results are reported as the mean ± sample standard deviation. SVM and KNN are deterministic for a fixed training set and were therefore evaluated once. For each seed, all metrics were computed from the same set of test predictions.

### 3.7. RLFormer Multimodal Framework

**Figure 9:**
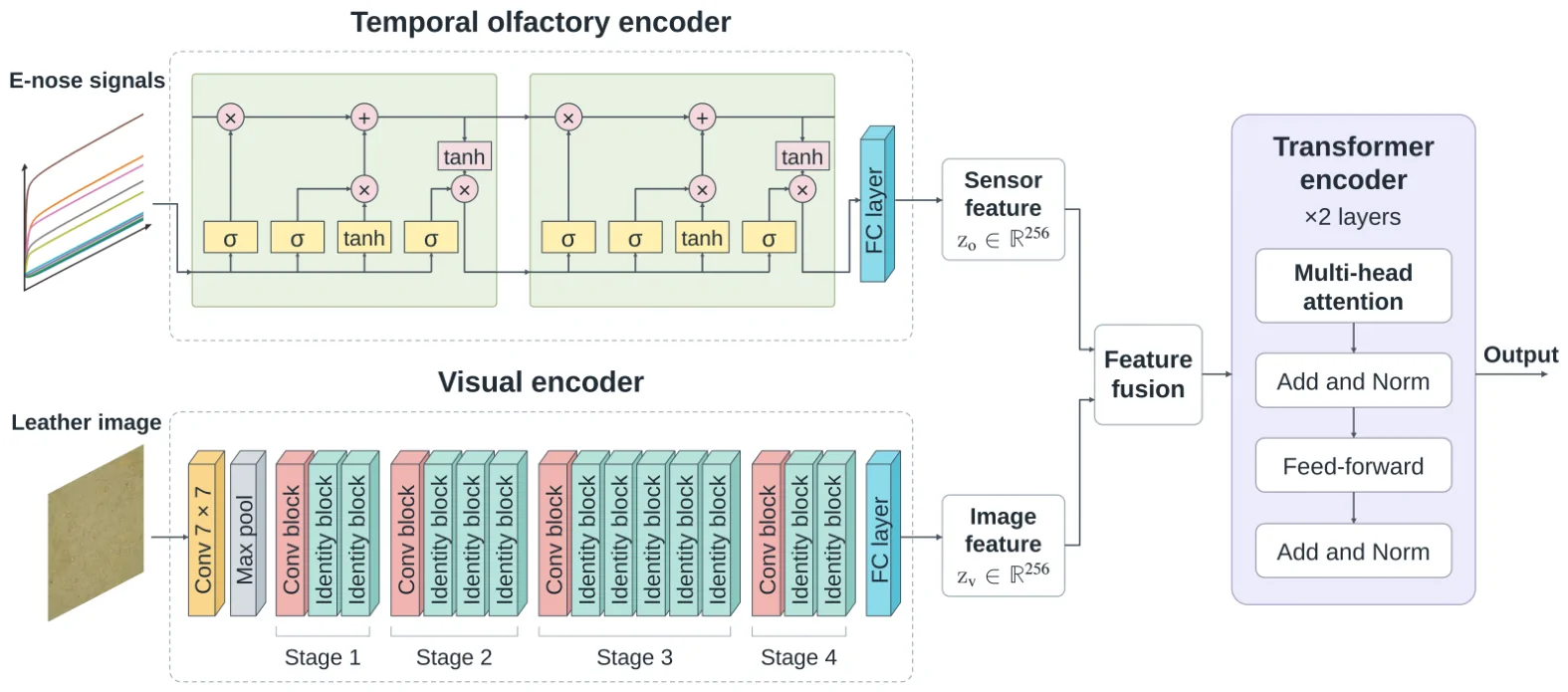
Schematic representation of the RLFormer framework.

RLFormer jointly models microscopic image features and E-nose response sequences. The two modalities describe complementary aspects of the specimens: microscopy captures the fine-scale surface texture, whereas the E-nose records the temporal evolution of a cross-responsive headspace fingerprint. RLFormer therefore uses modality-specific encoders followed by transformer-based interaction in a shared latent space. The architecture is shown in Figure 9.

#### 3.7.1. Visual Representation Learning

Each microscopic image was resized to 128×128 pixels and converted to grayscale. A ResNet50 backbone was used as the visual encoder [41]. In RLFormer, ResNet50 served as a feature extractor rather than as an independent classifier. The extracted feature was projected to a 256-dimensional embedding:

(12) $$z_{v}=P_{v}\;\left(\mathrm{ResNet}50(I)\right),$$

where *I* is the input image and $P_{v}\left(\cdot \right)$ denotes the visual projection layer. The resulting vector $z_{v}\in R^{256}$ was used for multimodal fusion.

#### 3.7.2. Temporal E-Nose Representation Learning

For each specimen, the E-nose input was represented as a 160×10 matrix containing 10 sensor channels sampled over 160 s. A two-layer long short-term memory (LSTM) network was used to model temporal dependencies [42]. The final sequence representation was projected to a 256-dimensional embedding:

(13) $$z_{o}=P_{o}\;\left(\mathrm{LSTM}(X)\right),$$

where *X* is the E-nose sequence and $P_{o}\left(\cdot \right)$ is the olfactory projection layer. The output $z_{o}\in R^{256}$ summarizes the temporal response fingerprint. No additional denoising was applied before sequence modeling in the reported experiments.

#### 3.7.3. Cross-Modal Interaction and Classification

The visual and E-nose embeddings were stacked to form

(14) $$H_{0}=\left[z_{v};z_{o}\right]\in R^{2\times 256},$$

where [·;·] denotes stacking. A two-layer transformer encoder was then used to model interactions between the two modality tokens. Multi-head attention enabled adaptive weighting of shared and complementary information, while residual connections, layer normalization, and feed-forward sublayers refined the representation. The interaction stage was written as

(15) $$H=\mathrm{Transformer}(H_{0}).$$

The fused representation was passed to a multilayer perceptron and softmax classifier:

(16) $$\hat{y}=\mathrm{Softmax}\;\left(\mathrm{MLP}(H)\right).$$

The methodological contribution is therefore the interaction-based integration of modality-specific representations rather than a new visual or recurrent backbone.

## 4. Conclusions

This study developed a non-targeted analytical workflow that integrates cross-responsive E-nose fingerprints with microscopic images for four-class leather authentication. Under a standardized 10-day acquisition design, the category-level daily mean E-nose responses had a global RSD of 6.18%; the variance-component analysis showed that this dispersion was dominated by a progressive between-day attenuation common to all four categories, whereas the within-day RSD averaged 3.81%. W1W, W2W, and W6S showed the largest sensor-wise class separation. The two modalities provided complementary information: direct ResNet50–LSTM fusion achieved a mean accuracy of 87.50±2.50%, while transformer-based interaction in RLFormer achieved a 97.67±2.16% accuracy and a Cohen’s kappa of 0.9689±0.0288 over five random seeds on specimens acquired during the final 3 days.

The findings establish a proof of concept for fusing volatile-response and microstructural fingerprints in leather screening. The reported classification performance reflects one controlled 10-day campaign and does not constitute compound-level identification or external deployment validation. Multi-lot sampling, compound-resolving analyses, drift management with reference measurements, and independent testing will be important for establishing analytical generalizability and practical robustness.

## Figures and Tables

**Table 1:** Variance components of the fixed-window E-nose responses obtained by a one-way random-effects ANOVA with acquisition day as the random factor; values are averaged over the four leather categories. ${\mathrm{RSD}}_{w}$, within-day RSD; ${\mathrm{RSD}}_{d}$, between-day RSD; ${\mathrm{RSD}}_{I}$, combined RSD; ICC, proportion of variance attributable to acquisition day; Trend, slope of the daily mean versus day expressed as a percentage of the category mean; $n_{\mathrm{sig}}$, number of categories (of four) with a significant linear trend (p<0.05).

Sensor	RSD_*w*_ (%)	RSD_*d*_ (%)	RSD_*I*_ (%)	ICC	Trend (%/Day)	$n_{\mathrm{sig}}$
W1C	1.69	4.74	5.04	0.89	+1.53	4
W5S	12.89	3.34	13.52	0.10	−1.44	3
W3C	1.20	4.75	4.90	0.94	+1.54	4
W6S	4.14	1.51	4.45	0.12	−0.19	0
W5C	0.64	2.87	2.95	0.95	+0.93	4
W1S	3.45	12.00	12.49	0.92	−3.88	4
W1W	3.50	8.04	8.77	0.84	−2.62	4
W2S	5.24	9.09	10.53	0.74	−2.57	4
W2W	2.38	8.17	8.52	0.92	−2.68	4
W3S	2.97	3.98	4.97	0.65	−0.62	0
Mean	3.81	5.85	7.61	0.71	—	31/40

**Table 2:** Performance of conventional models for E-nose-based leather classification on the chronological test set, evaluated at the observation level (2400 test observations). RF values are the mean ± sample standard deviation over five random seeds (42–46); SVM and KNN are deterministic and were evaluated once.

Model	Accuracy	Precision	Recall	F1-Score	Kappa
SVM	0.7092	0.7336	0.7092	0.7017	0.6122
RF	0.5046±0.0301	0.4941±0.0333	0.5046±0.0301	0.4727±0.0338	0.3394±0.0401
KNN	0.3475	0.3321	0.3475	0.3020	0.1300

**Table 3:** Performance of image-only convolutional models on the chronological test set. Values are the mean ± sample standard deviation over five random seeds (42–46).

Model	Accuracy	Precision	Recall	F1-Score	Kappa
CNN	0.8283±0.0869	0.8597±0.0518	0.8283±0.0869	0.8199±0.0998	0.7711±0.1159
VGG16	0.6667±0.0829	0.6828±0.0642	0.6667±0.0829	0.6588±0.0862	0.5556±0.1106
ResNet50	0.7733±0.0686	0.7541±0.1025	0.7733±0.0686	0.7403±0.0992	0.6978±0.0914

**Table 4:** Ablation results for unimodal, direct-fusion, and interaction-based model configurations on the chronological test set. Values are the mean ± sample standard deviation over five random seeds (42–46).

Model	Accuracy	Precision	Recall	F1-Score	Kappa
ResNet50	0.7733±0.0686	0.7541±0.1025	0.7733±0.0686	0.7403±0.0992	0.6978±0.0914
LSTM	0.6250±0.0250	0.6296±0.0216	0.6250±0.0250	0.6229±0.0223	0.5000±0.0333
ResNet50 + Transformer	0.7967±0.0326	0.8127±0.0227	0.7967±0.0326	0.7927±0.0315	0.7289±0.0435
LSTM + Transformer	0.7767±0.0224	0.7816±0.0194	0.7767±0.0224	0.7774±0.0225	0.7022±0.0298
ResNet50 + LSTM	0.8750±0.0250	0.8838±0.0200	0.8750±0.0250	0.8748±0.0256	0.8333±0.0333
Transformer	0.8200±0.0689	0.8327±0.0529	0.8200±0.0689	0.8183±0.0677	0.7600±0.0918
RLFormer	0.9767±0.0216	0.9783±0.0189	0.9767±0.0216	0.9765±0.0217	0.9689±0.0288

**Table 5:** Nominal sensitivity classes of the 10 MOS sensors in the PEN3 electronic-nose system. The classes describe broad response tendencies and should not be interpreted as compound-specific selectivity.

Sensor	Nominal Sensitivity Class	Broad Response Description
W1C	Aromatic compounds	Responsive to aromatic organic compounds
W5S	Nitrogen oxides	Broad response to nitrogen oxides
W3C	Ammonia and aromatics	Responsive to ammonia and aromatic compounds such as benzene
W6S	Hydrogen	Responsive to hydrogen
W5C	Alkanes and aromatics	Responsive to alkanes such as propane and to aromatic compounds
W1S	Methane	Responsive to methane
W1W	Sulfur compounds	Responsive to sulfur-containing compounds such as hydrogen sulfide
W2S	Alcohols, aldehydes, and ketones	Broad response to oxygenated organic compounds
W2W	Aromatics and organic sulfur compounds	Responsive to aromatic and organic sulfur-containing compounds
W3S	Alkanes	Responsive to alkanes such as methane

## Data Availability

The data presented in this study are available on request from the corresponding author. The data are not publicly available because they are part of ongoing research projects.
